# Fahr’s Disease: Case Presentation With Facial Numbness

**DOI:** 10.7759/cureus.43786

**Published:** 2023-08-20

**Authors:** Nimra Shahid, Ayodeji Dosu, Fazeen Nasser

**Affiliations:** 1 Medicine, University Hospital of North Tees, Stockton-on-Tees, GBR; 2 Internal Medicine, Wrexham Maelor Hospital, Wrexham, GBR

**Keywords:** movement disorders, visual disturbances, headache, seizures, sensory deficit, motor dysfunction, basal ganglia calcifications, hereditary neurological disorder, fahr's disease

## Abstract

Fahr's disease is a rare hereditary neurological disorder characterized by idiopathic basal ganglia and cerebral cortex calcifications. It presents a wide range of neurological manifestations, including motor dysfunction, sensory deficits, seizures, headaches, visual disturbances, and movement disorders.

We present a case report of a 42-year-old female who presented to the accident and emergency department with a stroke alert. Her main symptom was left facial numbness. Otherwise, she was fit and well. A CT scan of her head revealed significant bilateral basal ganglia calcifications rather than ischaemic or haemorrhagic changes. Blood tests showed normal serum calcium, normal phosphate, and normal parathyroid hormone levels. Upon further inquiry, she mentioned that her sister had been diagnosed with similar findings on a brain scan. Subsequently, an MRI scan of her brain was performed, which suggested Fahr's disease. Currently, there is no definitive management available, so a conservative management approach is usually employed based on symptomatology. This case is particularly interesting due to its rarity, strong genetic inheritance, and the development of a management plan.

## Introduction

Fahr's syndrome is an autosomal dominant, genetically inherited neurological disorder characterized by abnormal calcium deposition in different areas of the brain, including basal ganglia and the cerebral cortex. The presentation may vary from motor dysfunction, dysarthria, increased spasticity, seizures, sensory deficit, and parkinsonism [1]. This case report is about an incidental finding of bilateral basal ganglia calcification in a patient with sensory symptoms who was initially investigated for a stroke.

## Case presentation

**Figure 1:**
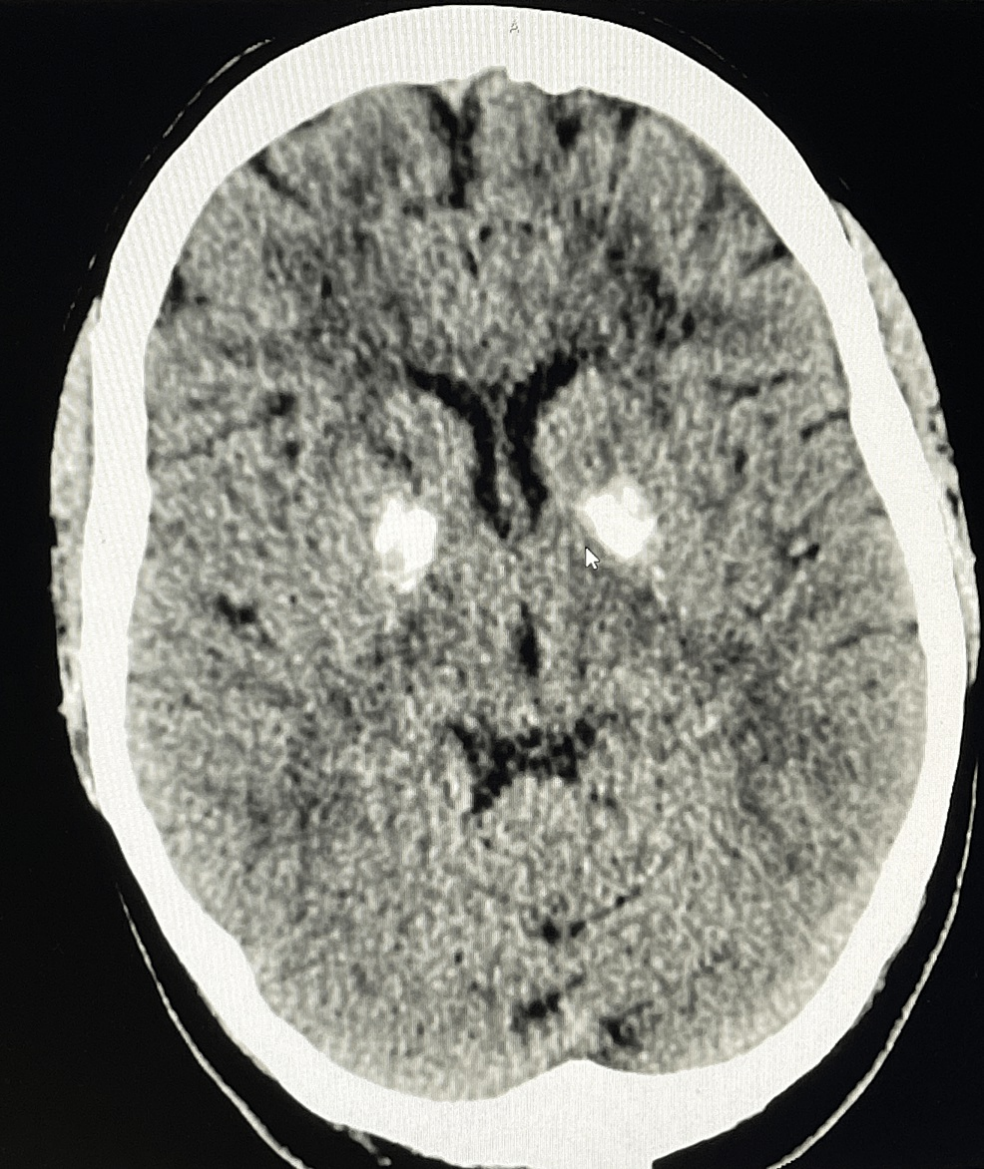
CT scan of the head showing bilateral basal ganglia calcification

We present a case of a 42-year-old female who presented to the accident and emergency department with acute onset of left facial numbness. She was having an odd feeling and discomfort in her left arm for a few months prior to this presentation. She was otherwise fit and well and had no significant medical history of note. Clinical examination showed she had reduced sensations on the left side of her face. No other sensory deficit was found. Tone, power, and reflexes in both upper and lower limbs were normal. No signs of cerebellar dysfunction were elicited, and cranial nerves examination was essentially normal. There was a concern that she may be suffering from a stroke. Hence, she had urgent imaging with a CT scan of her head, which revealed bilateral basal ganglia calcifications with no evidence of ischaemic or haemorrhagic change (Figure 1).

**Figure 2:**
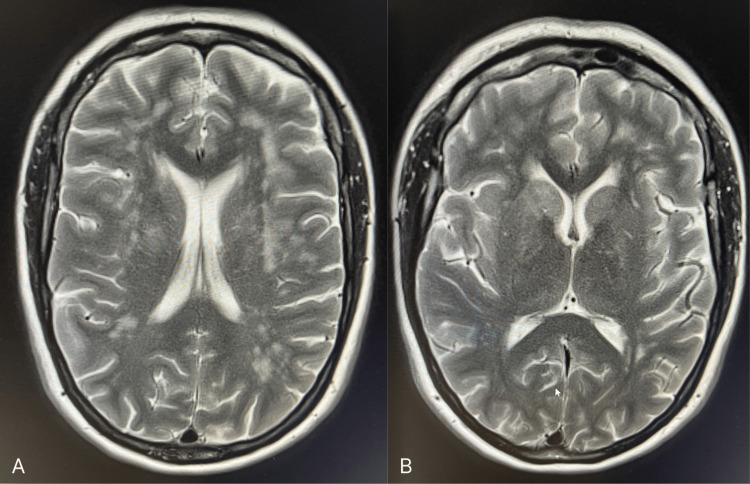
MRI scan of the brain showing white matter changes notable in the frontal lobes (A) as well as a signal dropout in the lentiform and caudate nuclei region (B)

She then proceeded to have an MRI of the brain, which also revealed florid high signal change within the white matter of both cerebral hemispheres, most notable in the frontal lobes as well as a signal dropout in the lentiform and caudate nuclei region on susceptibility-weighted imaging (Figure 2). These features are consistent with a diagnosis of suspected Fahr’s disease.

Blood tests, particularly serum calcium and serum parathyroid hormone levels, were within normal limits. On further inquiries about her family history, the patient reported that her maternal sister, who had also been diagnosed with Fahr's disease based on similar CT findings, reportedly presented with episodic headaches and tremors. However, detailed clinical information is unavailable. This further buttressed our point for reporting this case to increase awareness of this rare diagnosis. With her consent, our patient has agreed to have genetic testing done.

As no definite treatment exists for Fahr’s disease. She has been advised to have a healthy lifestyle and to inform her other siblings so they can have genetic screening as well.

## Discussion

First described by Karl Theodor Fahr in 1930 [2], Fahr’s disease, also known as striato-pallido-dentate calcifications or idiopathic basal ganglia calcification (IBGC), is a neurodegenerative disease with an autosomal dominant pattern of inheritance [3] and a single gene locus has been identified on chromosome 14q, although several other studies have identified other genetic mutations, suggesting a heterogenic pattern of inheritance [3]. One study done by Ding et al. identified a new SLC2OA2 mutation of IBGC, which was used to explore the association between different phenotypes and genotypes in Chinese patients with IBGC [4]. It is a rare neurodegenerative disorder characterized by bilateral and symmetrical calcium deposits, mainly in the basal ganglia, although there can also be calcium depositions in other areas of the brain. This is usually an incidental finding on a routine plain CT scan of the head while investigating for other neurological conditions [5], as in our index case.

Fahr’s disease is more common in males than females and usually manifests primarily after the age of 30 years [6]. Although it has been reported to be more common in males, our case underscores the variability and unpredictability of the disease's presentation. The manifestation of symptoms in both the patient and her sister, both females, emphasizes that gender differences, while statistically notable, are not absolute predictors of the disease.

Aetiology could range from metabolic disorders, such as hypoparathyroidism and pseudohypoparathyroidism [5], as reported by Basak RC, to infective, genetic, and neoplastic causes, hence clinical presentation is in turn variable. It can manifest as movement disorders, parkinsonism, memory decline, seizures, and neuropsychiatric symptoms [1]. It can also be asymptomatic.

Diagnosis is usually based on clinical presentation, evidence of symmetrical bilateral calcifications on imaging, and positive family history, although secondary causes should be excluded.

In our index case, our patient presented with left facial numbness and an odd sensation in her left arm. She did not describe any weakness in her arms or legs; however, a cranial CT scan was done to exclude a stroke, which eventually revealed abnormal but symmetrical calcifications in her basal ganglia. She also had an MRI of her brain (Figure 2), and the findings were consistent with suspected Fahr’s disease.

Further history from the patient and investigation findings, as described in our case, suggests that there is a genetic pattern in our index case, given the strong family history described, rather than a metabolic cause or any other infective aetiology that can cause secondary intracranial calcifications such as toxoplasmosis, cytomegalovirus, rubella, HIV, syphilis, or tuberculosis. Our patient has been offered genetic screening.

Treatment is largely conservative, as this is a progressive neurodegenerative disease, hence the underlying aetiology should be treated if a secondary cause is found. Genetic counselling and screening should be offered to other family members, as early diagnosis may be helpful in dealing with symptoms if they manifest early.

## Conclusions

Fahr’s disease is a neurodegenerative disorder with variable aetiology and symptomatology. CT scan of the brain is helpful for diagnosis and if there is a strong family history of Fahr’s disease, early genetic counselling and screening should be offered. There is no cure for this disorder currently.
